# Effect of repeated bolus and continuous doxorubicin administration on bone and soft tissue concentrations– a randomized study evaluated in a tumour-free porcine model

**DOI:** 10.1007/s00280-025-04768-7

**Published:** 2025-03-24

**Authors:** Andrea René Jørgensen, Mats Bue, Pelle Hanberg, Christina Harlev, Elisabeth Krogsgaard Petersen, Hans Christian Rasmussen, Jakob Hansen, Thomas Baad Hansen, Akmal Safwat, Maiken Stilling

**Affiliations:** 1 Aarhus Microdialysis Research Group, Orthopaedic Research Unit, Aarhus University Hospital, Aarhus N, Denmark; 2 Department of Clinical Medicine, Aarhus University, Aarhus N, Denmark; 3 Department of Orthopaedic Surgery, Aarhus University Hospital, Aarhus N, Denmark; 4 Department of Forensic Medicine, Aarhus University Hospital, Aarhus N, Denmark; 5 Department of Oncology, Aarhus University Hospital, Aarhus N, Denmark

**Keywords:** Doxorubicin, Microdialysis, Repeated administration, Tissue concentration

## Abstract

**Purpose:**

The aim of this study was to evaluate plasma and bone- and soft-tissue concentrations of doxorubicin following two administrations of either bolus or continuous infusion administered at a three-week interval. The achievement of adequate concentration at target sites is believed to be positively correlated to effect, and it has been suggested that concentrations are affected by the number of administrations.

**Methods:**

Eighteen female pigs were included in the study and randomized into two groups of nine receiving either a bolus or continuous infusion. The animals received a dosage of 2 mg/kg on day 1 and on day 22. From day 1 to 10, doxorubicin concentrations, as well as kidney and liver function, were monitored with plasma samples (total concentrations). On day 22, doxorubicin was measured in plasma samples (total concentration) and microdialysates (unbound concentrations) from subcutaneous tissue, muscle, synovial fluid of the knee joint, cancellous bone, and intravenously.

**Results:**

On day 22, the pharmacokinetic profiles were comparable between the two groups except for plasma AUC_0 − 12 h_, which was higher after continuous infusion, and intravenous C_max_, which was higher after bolus infusion. Bone- and soft tissue concentrations were below 0.10 µg/mL. Except for mean plasma (total) concentration at the 6 h timepoint on day 1 and 22 in the continuous group, which was higher after the first administration (*p* = 0.037), no differences in plasma concentrations were found between the two administrations.

**Conclusion:**

Low mean tissue doxorubicin concentrations and similar pharmacokinetic profiles were found between the bolus and continuous infusion groups. Thus, similar anti-neoplastic efficacy is to be expected with both administration types.

**Supplementary Information:**

The online version contains supplementary material available at 10.1007/s00280-025-04768-7.

## Introduction

Introduced clinically in the 1970s, the chemotherapeutic drug doxorubicin has been used for many decades to treat haematological, soft tissue and bone cancers including osteosarcoma [1]. Doxorubicin is traditionally administered intravenously as either bolus or continuous infusion. Continuous infusion attempts to lower the risk of cardiotoxicity, which is a feared and potentially fatal side-effect of doxorubicin treatment [2]. The lower incidence of cardiotoxicity seen after continuous infusion is believed to be caused by a lower plasma peak doxorubicin concentration [3, 4]. Doxorubicin can be administered as part of a multi-drug regime or as monotherapy, but when administered as monotherapy, the common dosage is 60–75 mg/m^2^ given every three weeks [5]. In the case of osteosarcoma, the conventional treatment relies on three key components: Neoadjuvant therapy, surgical removal of the tumour and courses of adjuvant therapy.

Despite being used clinically for decades, many aspects of the mechanisms of action of doxorubicin remain unclear. The effect is believed to be exerted intra- and extracellularly and at least partly positively correlated to exposure at target site [1, 6, 7]. Until recently, pharmacokinetic data on doxorubicin was based primarily on plasma concentrations, which may not always be a valid surrogate for the local target tissue concentrations due to variability in plasma-tissue exposure [8–10]. Studies assessing total plasma concentrations have found both inter- and intraindividual differences [11–13]. The interindividual differences may be attributed to body composition, patient age, gender, comorbidities, liver- and kidney function, drug combinations, tumour type, etc [13–22]. Moreover, the influence of the tumour microenvironment on treatment effect in individuals receiving same treatment with very varying results is also a popular topic of research. Moreover, the influence of the tumour microenvironment on variation in individual treatment efficacy also represents a compelling area of research. The microenvironment may also contribute to the emergence of drug resistance.

It has long been known that doxorubicin treatment causes a systemic inflammatory response, which is likely the cause of the intra-individual differences seen after repeated administrations [23]. Both higher and lower total plasma concentrations in the subsequent dosing interval have been described but the reasoning is unclear [11, 24–28]. Therefore, evaluation of target site concentrations of doxorubicin under varying pathophysiological conditions and following different dosing and administration scenarios is warranted to improve treatment outcomes.

Microdialysis is a catheter-based sampling technique that allows simultaneous and continuous sampling from tissues of interest [29]. Sampling is done of the unbound portion and thereby of the assumed active part of the drug of interest [29].

In a tumour-free porcine model, this study aimed to compare doxorubicin concentrations, with the use of microdialysis, in plasma and bone and soft tissues following two administrations of either bolus or continuous doxorubicin infusion administered at a three-week interval.

It was hypothesised that tissue concentrations would not be affected by the repeated administration.

## Materials and methods

### Study overview

Eighteen pigs were included and randomized into two groups of nine, receiving either two bolus or continuous administrations on day 1 and 22. On day 22, twelve hours of microdialysis sampling of doxorubicin bone and soft tissue concentrations was performed.

### Ethical approval

The study was conducted at the Department of Clinical Medicine, Aarhus University, Aarhus, Denmark. Approval was obtained from the Danish Animal Experiments Inspectorate (license No. 2021-15-0201-01079) and carried out in accordance with existing laws. All chemical analyses of doxorubicin were performed at the Department of Forensic Medicine, Aarhus University Hospital, Aarhus N, Denmark. Kidney and liver values were analysed by the Department of Clinical Biochemistry, Aarhus N, Denmark. The study was carried out in accordance with the ARRIVE-guidelines [30].

### Microdialysis

Microdialysis is a catheter-based sampling tool that allows for a dynamic and continuous sampling of molecules below the cut-off value of the semi-permeable membrane located at the tip of the catheter. The semi-permeable membrane allows concentration-dependent diffusion. As the catheter is connected to a perfusion pump continuously perfusing the catheter at a set flow rate, an equilibrium between the membrane and the surrounding extracellular space will not occur. This means that the concentration of the molecule of interest, quantified in the dialysate (collected in the collecting tube), only constitutes a fraction of the absolute concentration in the surrounding tissue. This fraction is referred to as the relative recovery, which must be calculated and corrected to estimate absolute tissue concentrations. In the present study, relative recovery was estimated using *retrodialysis by drug* with doxorubicin performed at the end of the study. Formulas for calculation can be found elsewhere [29, 31].

All microdialysis equipment was purchased from M Dialysis AB (Stockholm, Sweden). Based on results from a previous study, the equipment used for the collection of doxorubicin were the type 70 catheter with a 30 mm membrane and the type 67 intravenous catheter also with a membrane length of 30 mm (cut-off of 20 kDa) [32]. For all catheters, the flow rate was set at 1 µl/min, and the perfusion fluid was saline 0.9%. Sample collection was done in 1.5 ml LoBind Eppendorf tubes (Eppendorf, Hamburg, Germany) [32].

### Animals, sample size and randomization

Eighteen female pigs (Danish Landrace) were included in the study and randomized into two groups of nine. Randomization was done before the first intervention as block randomization of two (as one for the first two animals), by drawing a note indicating *Group 1* (bolus infusion) or *Group 2* (continuous infusion) from a non-translucent envelope.

Sample size was, due to a lack of relevant clinical assumptions based on results, from a study measuring doxorubicinol concentrations (metabolite of doxorubicin) in plasma as well as the hepatic system. Using a two-independent means power calculation with a mean doxorubicinol AUC (min×mM) of 3.8 (SD 1.4) in plasma (*n* = 4), a mean doxorubicinol AUC of 2.0 (SD 0.8) in the hepatic system (*n* = 4), a power of 90% and a significance level of 5%, a group size of *n* = 8 was found [4].

The animals arrived at the research facility a minimum of 14 days before the first intervention, providing sufficient time for acclimatization and human contact training. They were kept singularly in pens with a light cycle of 12 h. Straw was used as bedding, and the animals had access to water ad libitum. Feeding was restricted (farm pig ration) to control weight gain.

### Dosage

Doxorubicin dosage is traditionally administered based on body surface area. However, due to a lack of a suitable formula for the pig breed used in the present study, a dosage based on weight (2 mg/kg) was opted for. This approach has previously provided clinically relevant plasma concentrations [32]. Animals were fed to reach a mean weight of 66 kg on day 1 and 75 kg on day 22 resulting in dosages of 132 mg and 150 mg of doxorubicin, respectively. Pigs gain weight much faster than humans, wherefore, the dosage on day 22 had to be increased compared to the dosage on day 1. The actual attained mean weight was 65 kg on day 1 and 73 kg on day 22.

### Study set-up

#### Day 1

The first intervention indicated day 1 of the study period. The animals (mean weight: 65 kg) were sedated in their pens with zoletil mix ((25 mg/ml tiletamin + 25 mg/ml zolazepam) + 6.25 ml xylazine (20 mg/ml) + 1.25 ml ketamine (100 mg/ml) + 2.5 ml butorphanol (10 mg/ml) 1 ml/10 kg)) and placed under general anaesthesia with a combination of continuous intravenous infusion of propofol (Fresenius Kabi, Bad Homburg, Germany) and fentanyl (B. Braun, Melsungen, Germany). Initial dosages were 30 mL/h and 10–12 mL/h, respectively. A central venous catheter (CVC) was placed in the ear of each animal (size 4–6 F). This was not feasible for two animals, and insertion in the external jugular vein was used.

**Fig. 1:**
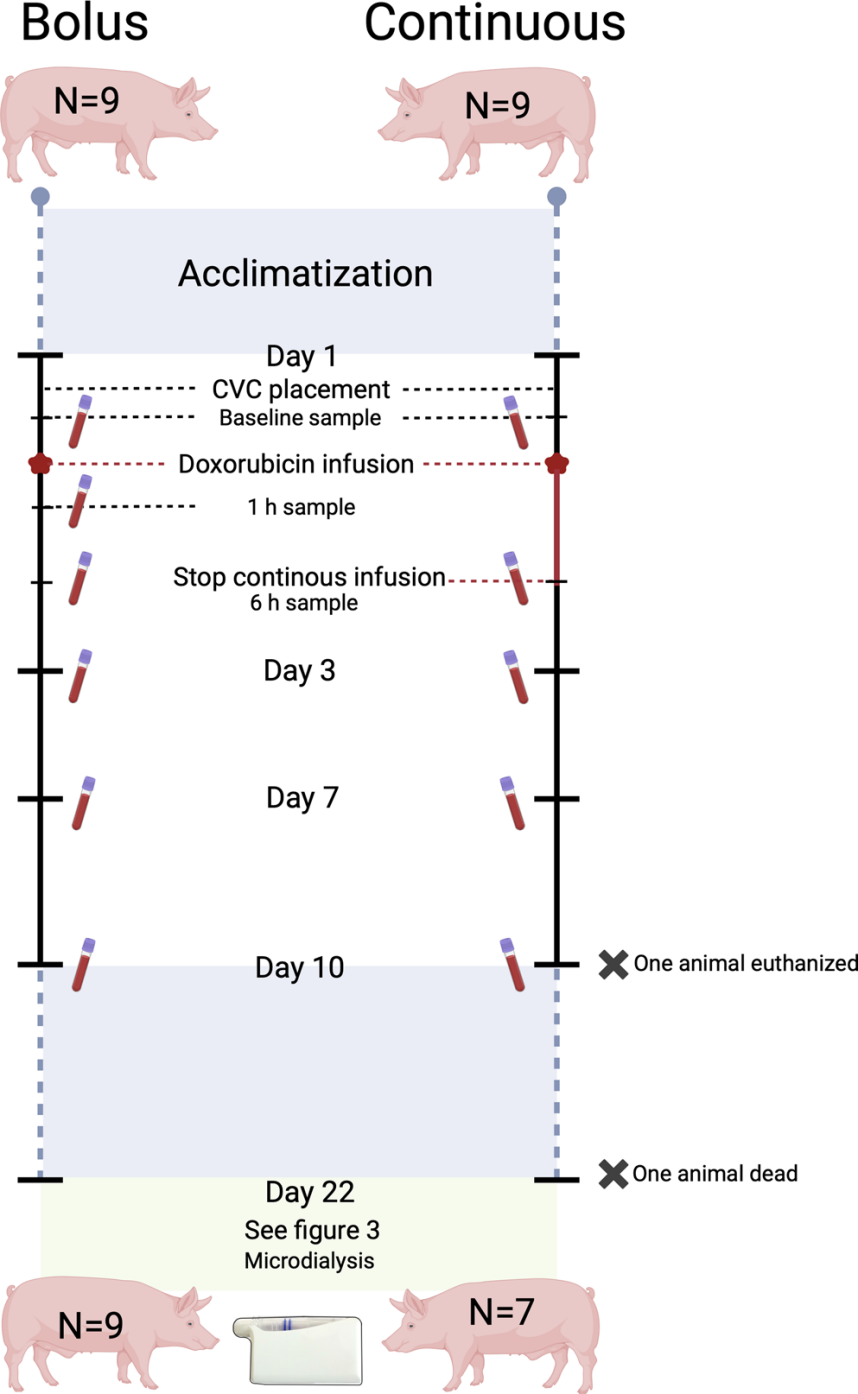
Overview of the sampling from day 1 until day 22. Created with Biorender

After placement of the CVC, baseline blood samples were drawn (Fig. 1). Hereafter, 500 mL of saline was administered through the CVC over 20–30 min, followed by administration of 132 mg doxorubicin soluted in 500 mL of saline 0.9%, given as either a bolus infusion over 5–15 min or a continuous infusion over 6 h. Blood samples were taken after 1 h (only bolus group) and 6 h. After doxorubicin infusion, an additional 100–200 mL saline was administered. All animals spent the same amount of time under anaesthesia (7–8 h) and were monitored with pulse, saturation and temperature. Animals were woken from anaesthesia and brought back to their in-house pens.

#### Day 2 till day 21; doxorubicin samples

Blood samples, for investigation of doxorubicin concentrations as well as liver and kidney status, were taken from the CVC on days 3, 7, and 10. The CVC was flushed daily with heparinized saline. After the blood sample on day 10, the CVC was removed. From day 11 to day 21, the animals were exposed to no further interventions.

All animals were observed several times daily and in the first days following the first doxorubicin administration scored on pulse, saturation, temperature, breathing, general behaviour, eating and defecation. In case of any signs of infection in terms of redness and swelling around the CVC, the ear was washed with chlorhexidine and fucidin ointment. Three animals were also treated with the antibiotic linco-spectin (i.m) due to signs of infection. Three animals were treated briefly with primperan on suspicion of nausea. Diarrhoea was a fairly common side effect a few days after doxorubicin treatment and was treated with vetmulin (i.m). A few animals had a decreased appetite in relation to the diarrhoea and were offered alternative food options. Symptoms of nausea/vomiting in combination with reduced well-being lasting more than 72 h meant exclusion from the study. All animals displayed a gradual increase in body weight up until day 22.

#### Day 22

**Fig. 2:**
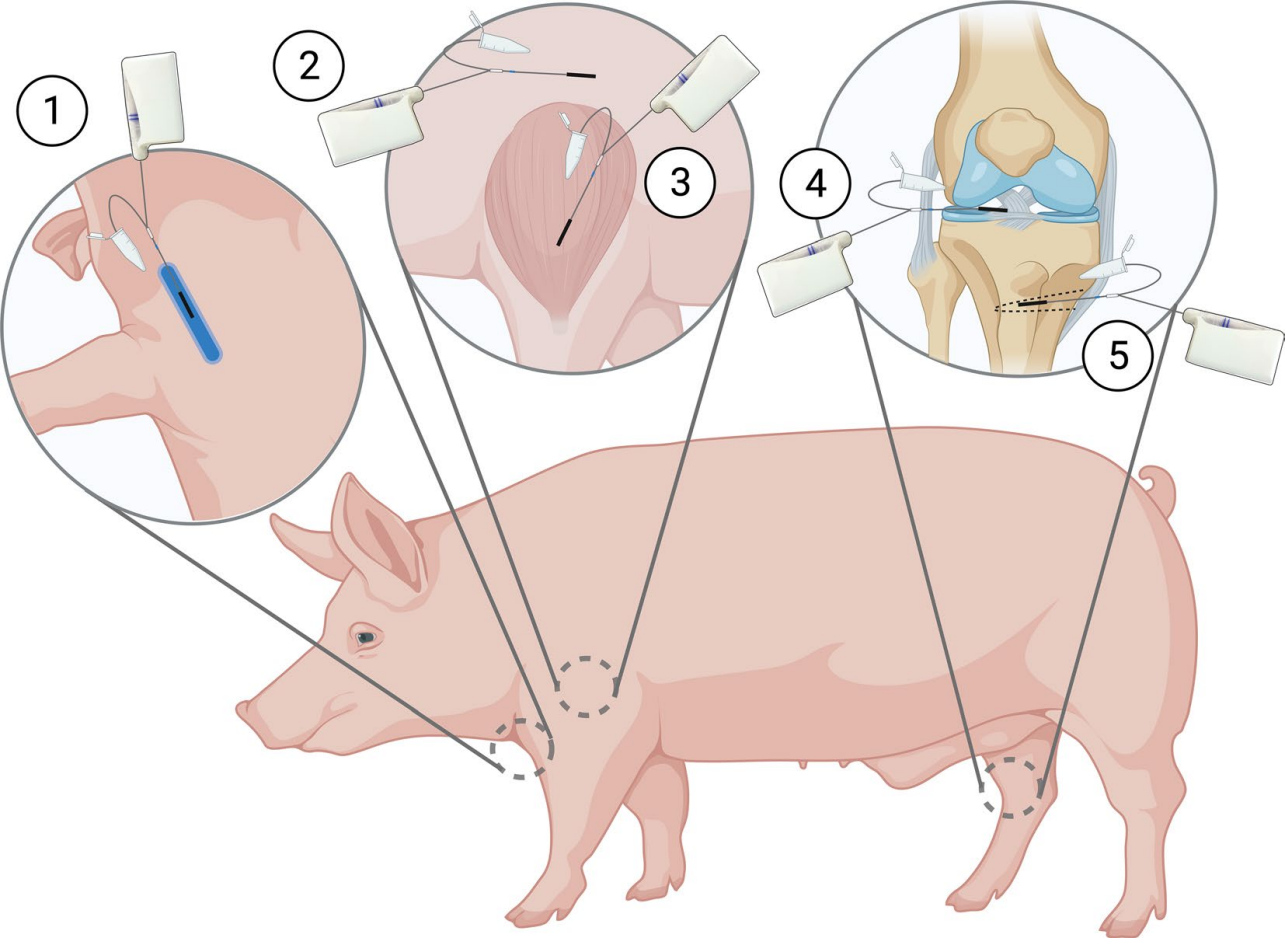
Placement of the microdialysis catheters on day 22. (1) Intravenous, (2) subcutaneous tissue, (3) muscle, (4) synovial fluid of the knee joint, and (5) cancellous bone compartments. Created with Biorender

The animals (mean weight: 73 kg) were sedated with zoletil mix and transported to the surgical facility. Upon arrival, animals were placed under general anaesthesia at initial dosages of 40 mL/h propofol and 25 mL/h fentanyl. By ultrasound guidance, two CVCs were placed in the external jugular veins and an arterial sheath in the groin. Microdialysis catheters were placed in four tissue compartments and one catheter intravenously, for measurement of unbound doxorubicin concentrations (Fig. 2). Muscle and subcutaneous catheters were placed on the right front limb using splitable introducers guided by ultrasound. An introducer was also used for the placement of a catheter in the synovial fluid of the knee joint. An incision on the medial side of the tibial tuberosity on the outer rotated right hind limb was made approximately 2 cm distal to the tibial plateau, continuing to the midpoint of the tibial diaphysis. A cancellous drill hole (35 mm in depth and ∅2 mm) was made in the metaphysis medial to the tibial tuberosity, and a catheter was placed. Bone overheating during drilling was prevented with frequent pausing and continuous cooling with saline. The position of catheters in synovial fluid of the knee joint and cancellous bone was verified intra-operatively with fluoroscopic imaging. Furthermore, the drill hole in the cancellous bone was verified post-mortem by computed tomography (CT). After placement, the microdialysis catheters were connected to perfusion pumps and filled with perfusion fluid (saline). Doxorubicin was administered according to group assignment on day 1, but 150 mg was administered due to increased body weight.

A minimum mean arterial pressure (MAP) of 65 mmHg was maintained to secure an equal circulation between the animals. In case MAP dropped below 65 mmHg and couldn’t be stabilized on fluids or in the Trendelenburg position, norepinephrine (concentration 0.1 mg/mL) was started at 0.3 mL/h and adjusted by response. Throughout the study time, the animals received a continuous infusion of 5% glucose to maintain glucose levels, which were monitored every two hours with arterial gasses including pH.

### Doxorubicin sampling day 22

**Fig. 3:**
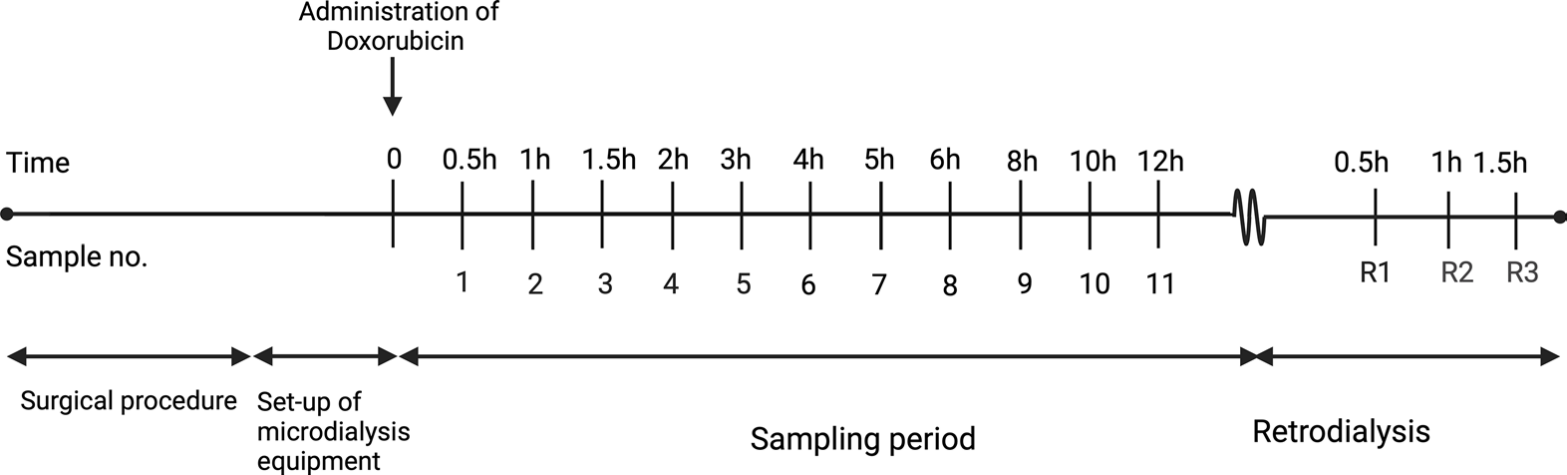
Overview of sampling of dialysates on day 22. Created with Biorender

The start of doxorubicin administration indicated time zero (Fig. 3). The overall sampling time was 12 h. The room light was off for the entire study period due to the risk of photodegradation of doxorubicin. Sampling was identical for animals receiving bolus and continuous infusion, respectively.

Dialysates were collected every 30 min from time 0 to 120 min, every 60 min from time 120 to 360 min, and every 120 min from time 360 to 720 min. A total of 11 dialysates were collected from each compartment plus an additional three retrodialysis calibration samples. Venous blood samples (total concentration) were taken at the midpoint of each of the above-mentioned sampling intervals and additionally after 60 and 360 min. Calibration was performed with an 18.74 µg/mL doxorubicin saline solution.

### Handling of samples

All venous blood samples taken for the quantification of the total concentration of doxorubicin was collected in EDTA 1.8 mg/mL 4 mL tubes and stored at 4–5 °C for a maximum of two hours. Samples were then centrifuged at 3,000 g for 10 min at 5 °C. Venous samples taken for the evaluation of kidney and liver status were collected in lithium heparin 5 mL tubes and centrifuged at 2,000 g for 10 min at 20 °C. All plasma samples and dialysates were immediately stored at -80 °C until analysis.

### Liver and kidney status

To ensure that variations in kidney and liver function between the two groups did not influence the results, a total of ten blood samples were taken from each animal. Samples were taken on day 1 before as well as 1 h and 6 h after the start of doxorubicin administration (time 0), respectively. One sample was taken on days 3, 7 and 10. On day 22, a sample was taken before as well as 1 h, 6 h and 12 h after the start of doxorubicin administration, respectively. Except for hemoglobin, all values were attained after analysis on an Atellica CH (Siemens Atellica Solution, Siemens Healthineers, Erlangen, Germany). Hemoglobin was analyzed on an ABL90 Flex Plus (Radiometer Medical Aps, Brønshøj, Denmark).

### Quantification of doxorubicin in microdialysates and plasma samples by ultra-high performance liquid chromatography and tandem mass spectrometry

The concentration of doxorubicin measured in dialysates represents the unbound concentration, while the concentration measured in plasma from venous samples is the total concentration. Doxorubicin concentrations in microdialysates/plasma were measured by ultra-high performance liquid chromatography and tandem mass spectrometry (UHPLC-MS/MS) as previously described in detail [32]. Briefly, microdialysate samples were prepared for analysis by mixing 10 µL dialysate with 190 µL internal standard solution (13CD3-doxorubicin stable isotope (Clearsynth, Mumbai, India) at 0.025 µg/mL in water: methanol (85:15) with 0.1% formic acid) in a 96-well plate (1mL Eppendorf LoBind). Blood plasma samples were prepared in 96-well plates (1 mL Eppendorf Lo-bind) by mixing 50 µL plasma with 50 µL saline (water with 0.9% sodium chloride) and 300 µL internal standard/protein precipitation solution (13CD3-doxorubicin at 0.05 µg/mL in 100% acetonitrile), followed by vortex-mixing and centrifugation (5000 ×g, 5 min). A 100 µL aliquot from the resulting supernatant was diluted with 200 µL water supplemented with 0.1% formic acid to yield the final sample. Separate samples for calibration were prepared in matched matrices at concentrations of 0, 0.001, 0.004, 0.012, 0.037, 0.111, 0.333 and 1 µg/mL using reference compound doxorubicin hydrochloride (European Pharmacopoeia Reference Standard CRS batch 7 supplied from Sigma-Aldrich, Germany). Samples were analyzed with an UHPLC-MS/MS system (Acquity UPLC coupled to a TQS mass spectrometer from Waters, Milford, Massachusetts, USA) with a C18 column (Waters UPLC HSS-C18, 1.8 μm, 100 × 2.1 mm) and using multiple reaction monitoring mode as described [32]. Calibration curves were constructed by linear regression of the peak area ratio (analyte/internal standard) versus the nominal analyte concentrations of the calibrant samples using 13CD3-doxorubicin as internal standard. The lower limit of quantification for doxorubicin was estimated to 0.002 (dialysate) and 0.003 µg/mL (plasma) and the standard requirements for precision (CV < 15%) and trueness (bias < 15%) were met.

### Pharmacokinetic analysis and statistics

For all animals and each compartment, the following pharmacokinetic parameters were determined for doxorubicin by non-compartmental analysis using Stata (version 16.0, StataCorp, College Station, Texas, USA). The area under the concentration-time curve (AUC_0 − 12 h_) from time zero until the end of the sampling period of 12 h was calculated using the linear up-log-down trapezoidal method. Peak drug concentration (C_max_) was calculated as the mean peak concentration of doxorubicin in each compartment. Penetration ratio was calculated by AUC_tissue_/AUC_iv_. For dialysates, all concentrations were assigned to the midpoint of each sampling interval.

The pharmacokinetic parameters for the animals receiving bolus infusion were compared to the animals receiving continuous infusion. The data was modelled separately for each compartment data to compare the groups (regression analysis with fixed effect) and analysed separately within groups to compare the compartments within groups (regression analysis with random effect). Mean plasma concentrations after 1 and 6 h on days 1 and 22 were compared in Excel (Microsoft version 16.78.3) using a t-test. A p-value < 0.05 was regarded as statistically significant. The p-values were not adjusted for multiple comparisons.

## Results

### Animals

Except for two animals receiving continuous infusion, all animals survived the entire study period. One animal was euthanized on day 10 due to continuous refusal to eat. The second animal died suddenly during the induction of anaesthesia on day 22. For both animals, all attained samples were included.

### Relative recoveries for Microdialysis

The ranges of mean relative recovery (SD) were 34% (8)– 80% (11) for the bolus group and 25% (9)– 79% (23) for the continuous group.

### Doxorubicin plasma concentration-time profiles day 1 to 22

**Fig. 4:**
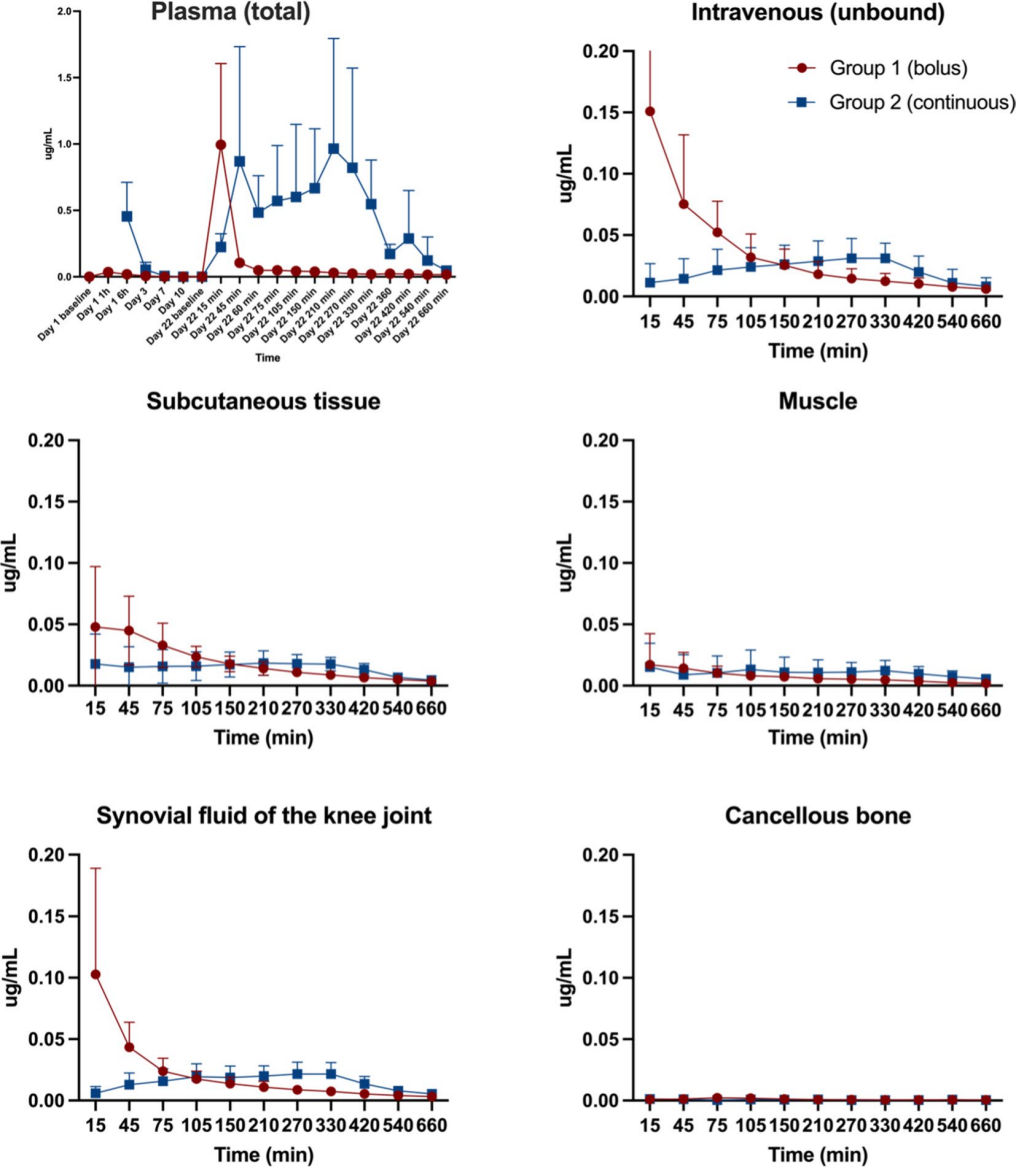
Mean time-concentration profiles (95% CI) of plasma (total) and the five microdialysis compartments (Intravenous, subcutaneous tissue, muscle, synovial fluid of the knee joint and cancellous bone). Bolus (red) and continuous infusion (blue). For the continuous group, there are no plasma concentrations for ‘Day 1 1 h’ as results could be unreliable as samples were taken through the same CVC as the infusion was administered. Baseline values for continuous infusion were 0 µg/ l Y-axis; doxorubicin concentration in µg/mL. Notice different values on the y-axis for plasma. X-axis; time in day, hours and min for plasma. Time in min for the microdialysis compartments

Figure 4 contains mean doxorubicin plasma (total) concentration-time profiles for both groups from day 1 to 22. On day 1, no clear peak was seen for the bolus group, probably because the first sample was taken after 1 h. For the continuous group, there are no plasma concentrations for ‘Day 1 1 h’ because the samples were taken through the same venous catheter as the infusion was administered– thus, reliable samples were not possible during doxorubicin infusion. Comparing the concentrations measured after 1 and 6 h after bolus administration on days 1 and 22, no intra-group differences were shown. For the continuous group after 6 h concentrations were higher after the first administration (*p* = 0.037).

### Doxorubicin concentration-time profiles day 22

Figure 4 also contains mean doxorubicin concentration-time profiles for each of the five microdialysis compartments. Individual concentration-time profiles for each animal on day 22 can be seen in supplementary Figures S1 and S2. A tendency of a prompt peak or a gradual increase was seen for all compartments after both bolus and continuous infusion. The exception is the intravenous (unbound concentration) compartment after bolus infusion, which seems heterogeneous, looking at the peak drug concentrations with a range of 0.02–0.41 µg/mL.

### Pharmacokinetic parameters on day 22 after bolus and continuous infusion; inter-group comparisons

Pharmacokinetic parameters for day 22 are presented in Table 1. Except for AUC_0 − 12 h_ for plasma (total concentration) in the continuous group and intravenous C_max_ in the bolus, which were significantly higher, no difference in AUC_0 − 12 h_ and C_max_ on day 22 was observed for the investigated compartments between the two groups. No difference was found between the two groups for AUC_tissue_/AUC_iv_. Mean C_max_ were generally very low, with values below 0.10 µg/mL. The only exceptions were plasma (total concentration) with mean C_max_ values of 0.82 and 0.65 µg/mL after bolus and continuous infusion, respectively. The intravenous compartment in the bolus group was the only compartment with a mean unbound C_max_ value of doxorubicin above 0.10 µg/mL (0.11 µg/mL).

**Table 1:** Pharmacokinetic parameters

Pharmacokinetic parameters	Bolus infusion		Continuous infusion		Difference (95%CI)	*p*-value
AUC_0 − 12 h_, min•µg/mL (95%CI)						
Plasma (total)	*N* = 9	40.3 (25.0; 55.5)	*N* = 5^a^	206.1 (117.1; 295.2)	165.9 (75.7; 256.2)	< 0.001*
IV (unbound)	*N* = 9	14.5 (9.0; 20.0)	*N* = 7	13.1 (7.4; 18.8)	-1.4 (-9.3; 6.5)	0.730
Subcutaneous tissue	*N* = 9	8.8 (4.6; 13.0)	*N* = 7	8.7 (4.9; 12.5)	0.1 (-5.7; 5.5)	0.972
Muscle	*N* = 9	3.6 (2.0; 5.3)	*N* = 7	6.5 (2.2; 10.9)	2.9 (-1.7; 7.5)	0.218
Synovial fluid of the knee joint	*N* = 9	8.7 (5.3; 12.1)	*N* = 7	9.4 (6.2; 12.7)	0.7 (-3.9; 5.4)	0.759
Cancellous bone	*N* = 9	0.5 (-0.5; 1.5)	*N* = 7	0.4 (-0.09; 0.9)	-0.1 (-1.2; 1.0)	0.858
**C**_**max**_, **µg/mL (95% CI)**						
Plasma (total)	*N* = 9	0.99 (0.34; 1.64)	*N* = 5^a^	0.82 (0.43; 1.21)	-0.17 (-0.93; 0.58)	0.646
IV (unbound)	*N* = 9	0.15 (0.06; 0.25)	*N* = 7	0.03 (0.02; 0.05)	-0.12 (-0.22; -0.02)	0.016*
Subcutaneous tissue	*N* = 9	0.05 (0.01; 0.10)	*N* = 7	0.02 (0.01; 0.04)	-0.03 (-0.07; 0.01)	0.177
Muscle	*N* = 9	0.02 (-0.01; 0.05)	*N* = 7	0.02 (0.01; 0.03)	0.0003 (-0.03; 0.03)	0.986
Synovial fluid of the knee joint	*N* = 9	0.11 (0.02; 0.20)	*N* = 7	0.02 (0.02; 0.03)	-0.09 (-0.17; 0.003)	0.059
Cancellous bone	*N* = 9	0.002 (-0.001; 0.005)	*N* = 7	0.002 (0.0004; 0.004)	-0.0002 (-0.004; 0.004)	0.990
**AUC**_**tissue**_ **/AUC**_**iv**_ **(95% CI)**						
Subcutaneous tissue	*N* = 9	0.66 (0.20; 1.12)	*N* = 7	0.92 (0.40; 1.44)	-0.25 (-0.95; 0.44)	0.460
Muscle	*N* = 9	0.28 (-0.19; 0.74)	*N* = 7	0.55 (0.03; 1.07)	-0.28 (-0.97; 0.42)	0.425
Synovial fluid of the knee joint	*N* = 9	0.67 (0.21; 1.13)	*N* = 7	1.20 (0.68; 1.72)	-0.53 (-1.22; 0.17)	0.132
Cancellous bone	*N* = 9	0.03 (-0.43; 0.49)	*N* = 7	0.03 (-0.49; 0.55)	-0.01 (-0.70; 0.69)	0.987

^a^Two animals died before and on day 22, and values from an additional two animals were excluded due to unreliable results

*Indicates statistical significance

AUC_0 − 12 h_: Area under the concentration-time curve; C_max_: Peak drug concentration

### Pharmacokinetic parameters on day 22 after bolus and continuous infusion; intra-group comparisons

AUC_0 − 12 h_ and C_max_ for plasma (total concentration) were higher than the other compartments after both continuous and bolus infusion (Table 2). Intravenous (unbound concentrations) were generally higher than the other compartments except the synovial fluid of the knee joint for both groups and the subcutaneous tissue after continuous infusion. Overall, the distribution to the different compartments was heterogeneous, with the lowest values of AUC_0 − 12 h_ and C_max_ found in the cancellous compartment for both groups.

**Table 2:** Comparison of AUC_0 − 12 h_ and C_max_ within groups

	Bolus infusion		Continuous infusion	
	AUC_0 − 12 h_	C_max_	AUC_0 − 12 h_	C_max_
IV vs. Synovial fluid of the knee joint	0.021*	0.409	0.228	0.154
IV vs. Muscle	< 0.001*	0.002*	0.003*	0.098
IV vs. Plasma	< 0.001*	0.003*	< 0.001*	< 0.001*
IV vs. Cancellous bone	< 0.001*	< 0.001*	< 0.001*	< 0.001**
IV vs. Subcutaneous tissue	0.033*	0.024*	0.042*	0.217
Synovial fluid of the knee joint vs. muscle	0.001*	0.021*	0.244*	0.644
Synovial fluid of the knee joint vs. plasma	< 0.001*	0.002*	< 0.001*	< 0.001*
Synovial fluid of the knee joint vs. cancellous bone	< 0.001*	0.005*	0.003*	< 0.001*
Synovial fluid of the knee joint vs. subcutaneous tissue	0.960	0.172*	0.770	0.939
Muscle vs. Plasma	< 0.001*	0.001*	< 0.001*	< 0.001*
Muscle vs. Cancellous bone	< 0.001*	0.093	0.002*	0.003*
Muscle vs. Subcutaneous tissue	0.003*	0.093	0.068	0.628
Plasma vs. Cancellous bone	< 0.001*	< 0.001*	< 0.001*	< 0.001*
Plasma vs. Subcutaneous tissue	< 0.001*	0.001*	< 0.001*	< 0.001*
Cancellous bone vs. subcutaneous tissue	< 0.001*	0.004*	< 0.001*	< 0.001*

*Indicates statistical significance (< 0.05)

AUC_0 − 12 h_: Area under the concentration-time curve; C_max_: Peak drug concentration

### Liver and kidney status

Supplementary Fig. 3 shows the time-concentration curve for nine different markers of liver and kidney status, which appears comparable between the two groups. Potassium, calcium and creatinine seem to be highly affected. However, the similar profiles indicate that both groups were affected equally by the doxorubicin administration. Comparing the results for the time of the two administrations, they were similar except for creatinine and sodium, which seemed much higher and lower at the end of day 22.

## Discussion

The aim of this study was to explore plasma, bone, and soft tissue concentrations after two doxorubicin administrations of either bolus or continuous infusion at a three-week interval. The main finding was low tissue concentrations, with minimal differences in day 22 AUC_0 − 12 h_, AUC_tissue_/AUC_iv_ and C_max_ when comparing bolus and continuous administration. Differences proving to be potentially clinically relevant were a higher AUC_0 − 12 h_ for plasma (total) after continuous infusion as well as a higher intravenous (unbound) C_max_ after bolus infusion.

This suggests that both the bound and unbound fractions exhibit a certain degree of dependence on the mode of administration. This underlines the necessity of evaluating target site exposure following all pertinent administration routes. Doxorubicin has both intra- and extracellular mechanisms of action, and it could be beneficial to explore this diffusion balance further to understand the factors influencing it.

### Clinical target

The effect of doxorubicin treatment is based on clinical and radiological evaluations, and no correlated pharmacokinetic targets exist. The closest to an effect parameter is the tumour cell line half-maximal inhibitory concentration (IC50), which is defined as the concentration capable of inhibiting 50% of a tumour cell line. However, IC50 is defined in vitro and can, therefore, not uncritically be extrapolated to a clinical setting. The lowest IC50 value for an osteosarcoma cell line is 0.017 µg/mL, which is higher than the C_max_ values found in cancellous bone after both administration forms on day 22 [33]. Additionally, the time needed above or equal to IC50 is also unknown.

Measuring the target intra-tumoral concentration therefore seems to be of utmost interest. Microdialysis presents itself as a usable tool for intra-tumoral measurements, and the risk of tumour cell seeding after implantation of the microdialysis catheter has been estimated to be similar to that of fine needle biopsies of < 0.005% [34, 35]. With the use of microdialysis, Müller et al. evaluated the concentrations of the chemotherapeutic drug 5-Fluororacil intratumorally and in nearby healthy subcutaneous tissue in 10 patients with breast cancer during their first cycle of neoadjuvant chemotherapy [6]. They found a correlation between intra-tumoral AUC and clinical tumour response. In comparison, a correlation could not be found when looking at plasma or subcutaneous AUC. Interestingly, the plasma AUCs for the non-responders in the study by Müller et al. were some of the highest values found [6]. This may be caused by differing tumour vascularisation and microenvironment.

Doxorubicin is known to cause systemic inflammation affecting all cells of the body, which potentially could also affect the penetration into the tumoural tissue. The level of intratumoural inflammation could be evaluated with the use of microdialysis, and a correlation to clinical effect could be investigated.

### The effect of repeated administrations

Doxorubicin is administered several times during treatment, but clinical studies have reported that repeated administrations impact the plasma concentrations [24–26, 28]. Two clinical studies (*n* = 45, *n* = 12) reported an increase in plasma concentration, whereof one of them attributed it to a decrease in the central volume of distribution [25, 26]. This is contradicted by two other clinical studies (*n* = 19, *n* = 15) finding decreased plasma concentrations [24, 28]. None of the studies had investigated effects in tissue.

Comparing mean plasma concentrations measured after 1 and 6 h, on day 1 and day 22, in the present study, the only significant difference was found in the continuous group after 6 h. However, this may very likely be due to a small sample size for these measurements (*n* = 6). When comparing the tissue data found on day 22 in the present study with a previous study performed in our group measuring the tissue concentrations on day 1 in a comparable animal group of 16 pigs (8 receiving continuous infusion and 8 receiving bolus infusion with the same dosage regime of 150 mg doxorubicin) only minor differences are seen [31].

### Inter- and intraindividual differences

Many clinical studies have found a high level of inter- and intraindividual differences in doxorubicin plasma (total concentrations) pharmacokinetics [11, 27]. This could be caused by heterogeneous study populations due to varying cancers, liver- and kidney function, age, etc. In the study population evaluated in the present porcine study, the liver- and kidney function was equal, and normal, between the two groups and between days 1 and 22 [36]. This observation provides reassurance that variations in results between the two groups cannot be attributed to differences in these organ functions. The only exceptions were creatinine and sodium, which increased and decreased (within the normal range), respectively, at the end of day 22 compared to day 1. However, with the low fraction excreted through the kidneys (5–15%) as well as the overall short amount of time for which this occurred, the clinical relevance seems small [5, 12, 37, 38].

Looking at the blood compartments (plasma; total concentration and intravenous; unbound concentration), a tendency of higher variance in the time-concentration profiles was seen for intravenous in the bolus group, which consonants with the findings of high intra- and interindividual concentrations in previous studies of only the plasma compartment [11, 24–28].

### Limitations

None of the animals in the present study had a tumour, wherefore any potential effects due to tumour tissue on the doxorubicin concentration after repeated administration could not be evaluated. Pigs are generally considered a good experimental model due to their anatomical and physiological resemblance to humans [39]. Also, the animals in the present study were 5 months of age with an active growth plate, which potentially could affect the translational potential of the concentrations to adult bone. During the study, the animals also gained a lot of weight, which follows the animals’ natural growth, but could potentially affect the distribution. However, for other drugs (antibiotics), a similar PK profile in bones between humans and young pigs has been found following continuous infusion [40, 41].

## Conclusion

After two consecutive administrations of either bolus or continuous infusion of doxorubicin, low tissue and intravenous (unbound) concentrations, but similar pharmacokinetic profiles, were found between the two groups on day 22. The only differences were a higher plasma (total) AUC_0 − 12 h_ found in the continuous group and a higher intravenous (unbound) C_max_ found in the bolus group. However, the differences may not be enough to have any clinical significance. In this very homogenous study population, no apparent signs of changing concentrations or large pharmacokinetic intra- and inter-individual differences were seen after two administrations.

## Electronic supplementary material

Below is the link to the electronic supplementary material.

Supplementary Material 1

## Data Availability

No datasets were generated or analysed during the current study.
