# Supplementary material for: Effect of repeated bolus and continuous doxorubicin administration on bone and soft tissue concentrations– a randomized study evaluated in a tumour-free porcine model
Source: Cancer Chemother Pharmacol. 2025 Mar 24;95(1):47. doi: 10.1007/s00280-025-04768-7 (PMC11930866; doi:10.1007/s00280-025-04768-7)
Supplement: Supplementary file 1 — Supplementary Material 1 [file 280_2025_4768_MOESM1_ESM.docx]

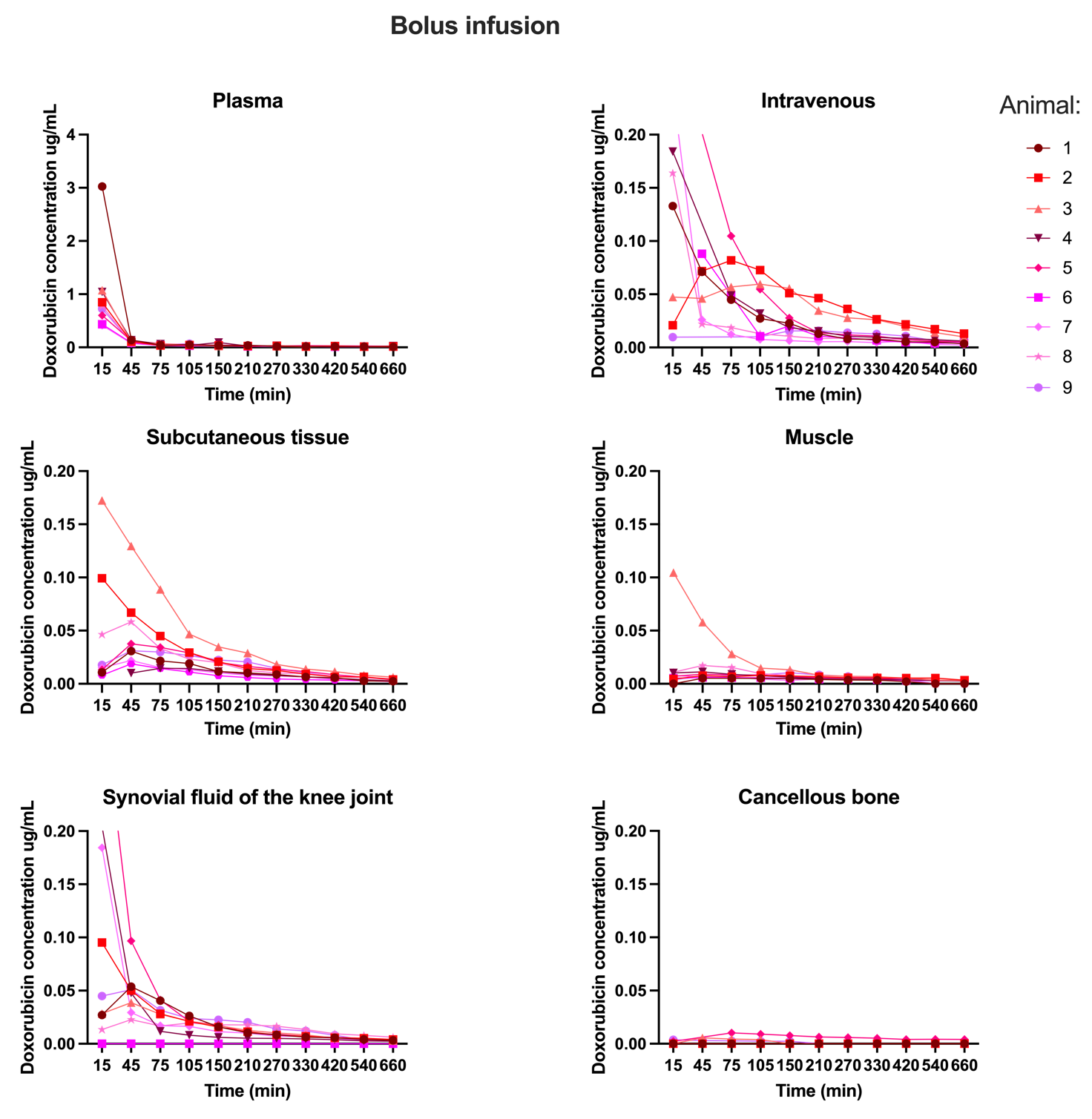


Supplementary 1: Individual time-concentration profiles (95% CI) for each animal receiving bolus infusion. Y-axis; doxorubicin concentrations in μg/mL. The y-axis for plasma differs from the remaining graphs. X-axis; time in minutes. Intravenous; animal 5 has a C_max_ of 0.41 μg/mL and animal 7 has a C_max_ of 0.24 μg/mL. Synovial fluid of the knee joint: animal 4 has a C_max_ of 0.20 μg/mL and animal 5 has a C_max_ of 0.33 μg/mL.


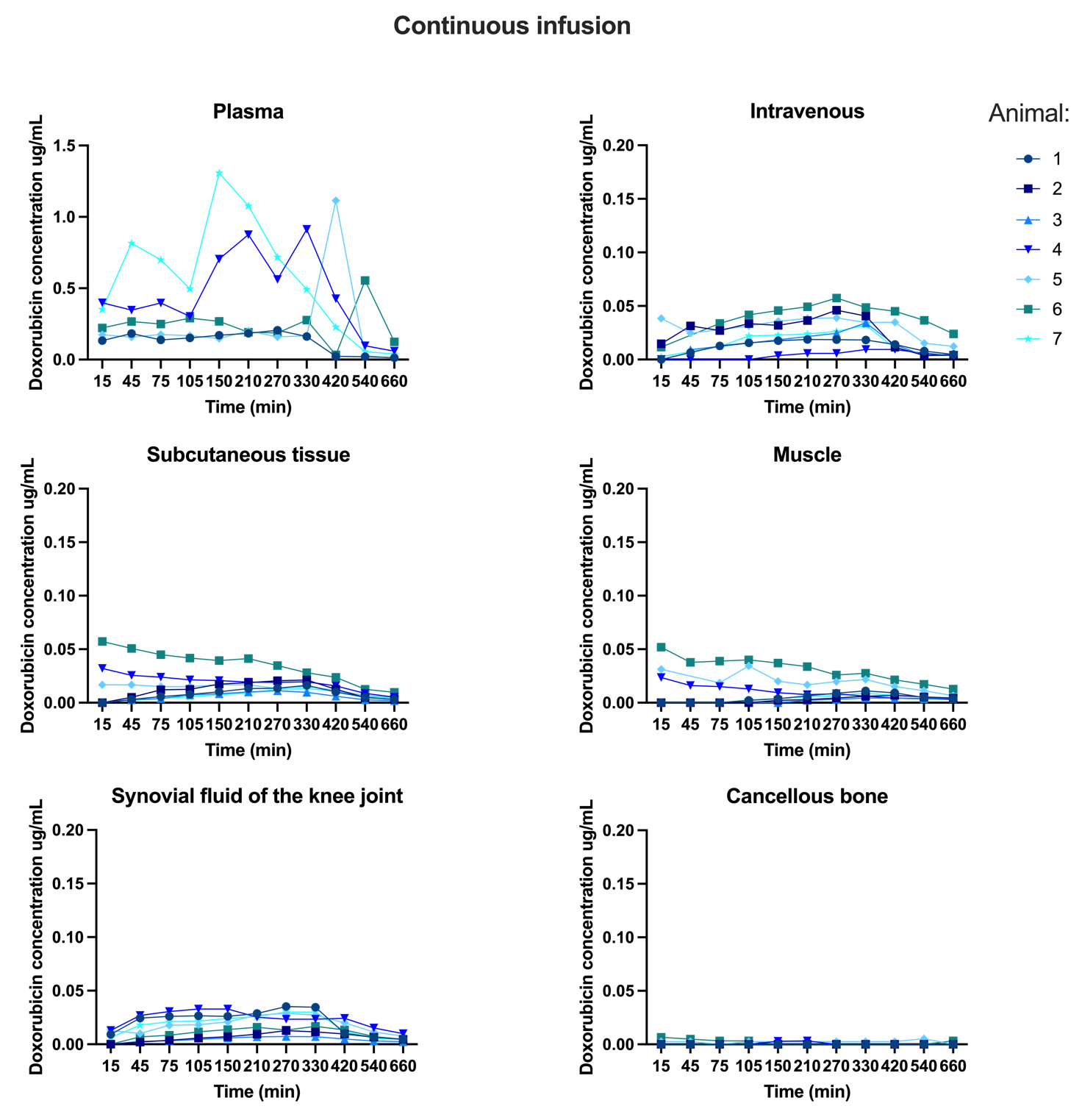


Supplementary 2: Individual time-concentration profiles (95% CI) for each animal receiving continuous infusion. Y-axis; Doxorubicin concentrations in μg/mL. The y-axis for plasma differs from the remaining graphs. X-axis; time in minutes

*
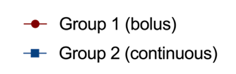
*
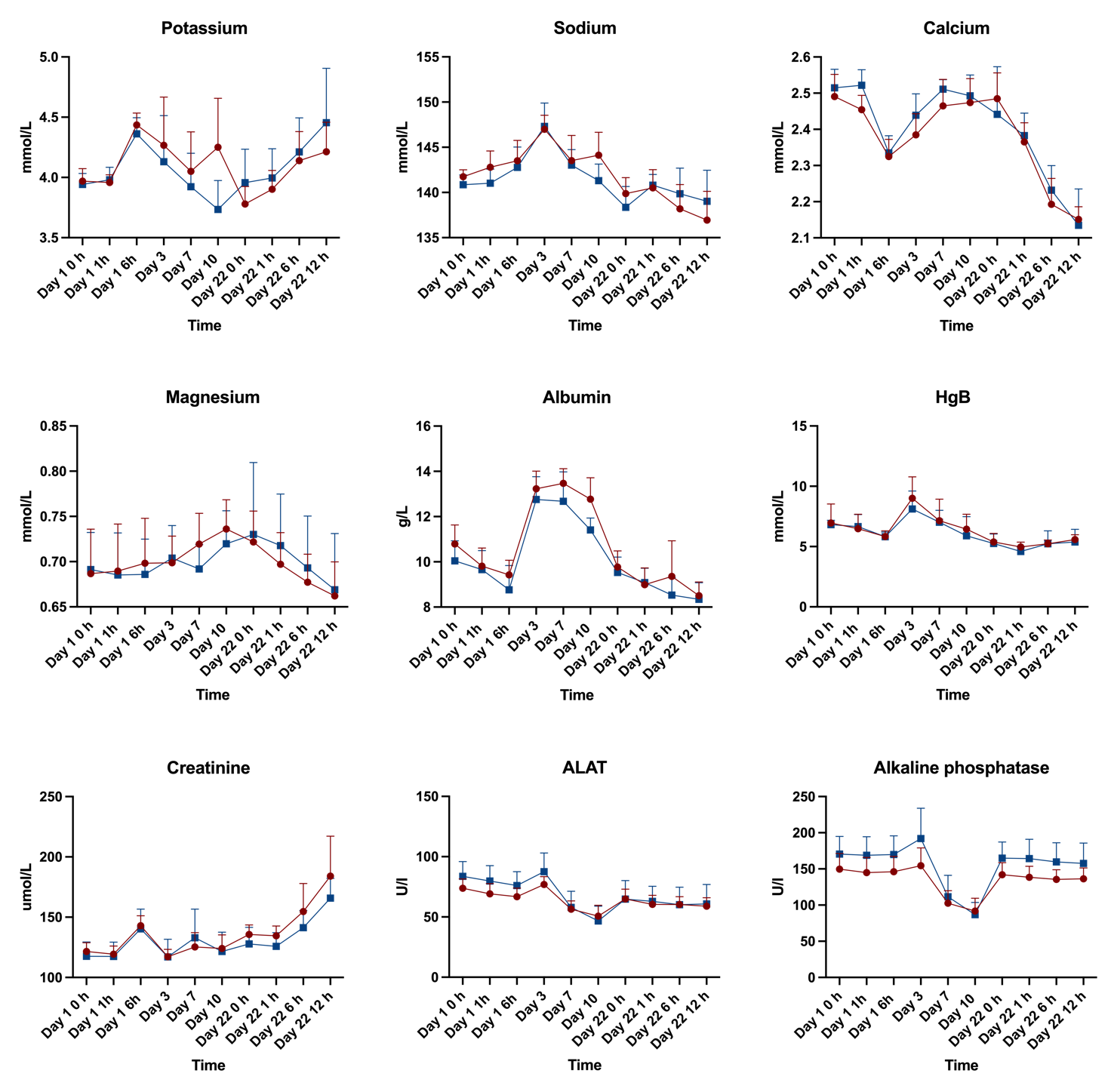


Supplementary 3: *Liver and kidney status (95%CI). Bilirubin was also measured but was below LLOQ (3 μmol/L) for all animals, except one in the bolus group (4.9-6.3 μmol/L) and two in the continuous group (4.3 and 3.9-4.9 μmol/L). CRP was also measured and was below LLOQ (4 mg/L) for all animals except one animal in the continuous group, which had a single measurement of 4.47 mg/L. Y-axis; concentration in mmol/L, g/L, μmol/L and U/l. X-axis; time in days and hours.*
